# Leptin-Induced JAK/STAT Signaling and Cancer Growth

**DOI:** 10.3390/vaccines4030026

**Published:** 2016-07-26

**Authors:** McKay Mullen, Ruben Rene Gonzalez-Perez

**Affiliations:** Department of Microbiology, Biochemistry and Immunology, Morehouse School of Medicine, Atlanta, GA 30310, USA; mmullen@msm.edu

**Keywords:** AK, STAT, leptin, cancer, NILCO, JAK/STAT inhibitors

## Abstract

Growth factor and cytokine signaling can influence the development of several cancer types. One of the key players in the development of cancer is the Janus kinas (JAK) signal transducer of activators of transcription (STAT) signaling pathway. The majority of growth factors and cytokine interactions with their membrane-bound receptors trigger JAK-STAT activation. The influential relationship between obesity and cancer is a fact. However, there is a complex sequence of events contributing to the regulation of this mechanism to promote tumor growth, yet to be fully elucidated. The JAK-STAT pathway is influenced by obesity-associated changes that have been shown to impact cancer growth and progression. This intricate process is highly regulated by a vast array of adipokines and cytokines that exert their pleiotropic effects on cancer cells to enhance metastasis to distant target sites. Leptin is a cytokine, or more precise, an adipokine secreted mainly by adipose tissue that requires JAK-STAT activation to exert its biological functions. Leptin is the central regulator of energy balance and appetite. Leptin binding to its receptor OB-R in turn activates JAK-STAT, which induces proliferation, angiogenesis, and anti-apoptotic events in normal cells and malignant cells expressing the receptor. Leptin also induces crosstalk with Notch and IL-1 (NILCO), which involves other angiogenic factors promoting tumor growth. Therefore, the existence of multiple novel classes of therapeutics that target the JAK/STAT pathway has significant clinical implications. Then, the identification of the signaling networks and factors that regulate the obesity-cancer link to which potential pharmacologic interventions can be implemented to inhibit tumor growth and metastasis. In this review, we will discuss the specific relationship between leptin-JAK-STAT signaling and cancer.

## 1. Introduction

The Janus kinase-signal transducer and activator of transcription (JAK/STAT) signaling pathway is an important hub for the regulatory actions of cytokines and growth factors. Data from many laboratories strongly suggest that leptin, one of the main adipokines secreted by the adipose tissue, plays an important role in the increased incidence, growth, recurrence and chemoresistance of several cancer types. The essential initial event triggered by leptin binding to its receptor, OB-R, is JAK/STAT activation, which is a current target for cancer treatment [1]. The obesity epidemic is extending to developing countries and is believed to reflect factors linked to dietary habits and physical activity at both the individual and societal levels [2]. This is due to increased availability of inexpensive calorie-dense foods, larger portion sizes and caloric intakes, reduced physical activity programs in schools, the adoption of a more sedentary lifestyle as well as increased screen time (i.e., watching television, using computers and mobile devices) [3,4]. Large population-based prospective studies have demonstrated consistent increased cancer incidences per 5 kg/m² increase in BMI [2]. It is this reason that the suppression of tumor specific growth factors could provide new insight into novel forms of therapy aimed at eliminating cancer with minimal risk to the overall health of the patient. Immunotherapeutic strategies to reset the balance between pro- and anti-inflammatory cells could also be crucial to controlling immune control, attenuating obesity-associated inflammation and treating/preventing resultant disease [2].

An association of obesity with cancer is biologically plausible because adipose tissue is metabolically active, secreting estrogens, adipokines (e.g., leptin), and cytokines [3]. Obesity is characterized by chronic inflammation and abnormal cytokine profiles [5]. Leptin is a 16-kDa peptide hormone and inflammatory cytokine involved in regulating food intake, metabolism, body fat, energy expenditure and neuroendocrine function [6]. Leptin secretion is gender dependent. Women show three-fold higher leptin levels than men, which is likely due to androgen actions and adrenocorticotropic hormone [2]. Leptin levels are elevated in overweight and obese individuals who develop leptin-resistance, a condition characterized by the inability of leptin to control appetite or energy balance. Research has demonstrated that the factors secreted in response to obesity have a strong influential role in promoting the growth and metastasis of cancer cells [6]. Placental tissue secretes leptin, which is a proliferation factor for the growing embryo. Additionally, low secretion of leptin has been detected in non-adipose tissue (i.e., stomach, skeletal muscle, brain, placenta and endometrium at the time of embryo implantation [7]. Remarkably, leptin/OB-R signaling is linked to the progression of cancers from breast, endometrium, pancreas, bladder, brain, colon, kidney, esophageal, lung, liver, prostate ovarian, skin, and thyroid cancers [8].

## 2. JAK/STAT Pathway

The JAK/STAT pathway is one of the architecturally simplest paradigms, allowing direct communication from transmembrane receptors to the nucleus [9]. The JAK/STAT pathway is now recognized as an evolutionarily conserved signaling pathway employed by diverse cytokines, interferons, growth factors, and related molecules [9]. The conservation of this pathway across species illustrates the inherited level of significance and importance that this mechanism embodies with regard to the signaling of growth development, differentiation, and cellular proliferation. In mammals, the JAK family consists of four members: JAK1, JAK2, JAK 3 and Tyk2. JAK stimulation occurs upon ligand-mediated receptor multimerization that induces close spatial proximity of two JAKs, allowing their trans-phosphorylation and trans-activation [10]. The JAK/STAT pathway can be activated by various mechanisms, including autocrine/paracrine cytokine production, activating mutations of receptors, JAKs or other upstream oncogenes that in turn activate STATs (e.g., EGF, HGF), and activating mutations of STATs themselves [11].

There are seven identified members of the STAT transcription factor family: STAT 1through 4, 5A, 5B, and 6 [12]. Attenuated STAT1 expression during adipogenesis of human adipocytes suggests that it may play a role in the transcriptional regulation of this process [13]. Recent research suggests that crosstalk with other signaling pathways, combined with the inflammatory state of adipose tissue, are essential factors influencing the ability of STAT1 to regulate adipogenesis [13]. A summary of the identification of STAT target genes in adipocytes reveals how these transcription factors affect multiple areas of adipocyte metabolism, including glucose homeostasis, insulin action, and modulation of lipid stores [13].

Studies show that many STAT activators play an important role in facilitating adipocyte gene expression and exhibiting differential expression in conditions of obesity and/or insulin resistance [14,15]. Particular STAT proteins play a dedicated role in mediating signaling by defined subgroups of cytokine receptors [16]. Cells and tissues of STAT1 deficient mice display a generalized state of unresponsiveness to either IFNγ or IFNα when tested either under well-controlled in vitro conditions or under physiologic in vivo conditions in intact mice [16]. Activation of STAT1 and STAT3 leads to target gene transcription, making these factors essential drivers of cellular proliferation [17]. Aberrant STAT activation has been found in multiple types of tumors, showing implications in the parthogenesis of diffuse large B cell lymphoma as well as solid-organ malignancies, such as breast and nasopharygeal carcinoma [11].

Other STAT proteins, such as STAT5A and STAT5B, are members of a well-known group of transcription factors [12]. During normal mammary gland development, STAT5A plays the more dominant role, whereas both STAT5A and STAT5B have been described as contributing to breast cancer pathophysiology [12]. Although STAT6 is abundantly expressed in preadipocytes as well as throughout fat cell differentiation, its activators, functions, and target genes remain yet to be fully elucidated [13].

Members of the avian erythroblastosis virus E26 onocogene (ETS) family of transcription factors have a distinct role in regulating cellular proliferation and differentiation, from embryonic development well into maturity due to constitutive JAK/STAT signaling [18]. However, deregulation of this transcription factor through genetic alteration often leads to tumorigenesis [18]. The JAK/STAT signaling pathway explains a great deal of cytokine biology and identification of the disease-causing mutations (i.e., breast cancer) indicating that this mechanism may have major therapeutic implications [13].

## 3. Leptin-Induced Activation of the JAK/STAT Pathway and Cancer

Leptin binds the leptin receptor, OB-R, a type I cytokine receptor that lacks autophosphorylation capabilities and shows no intrinsic kinase activity, thus requiring an auxiliary kinase (JAK) to initiate signaling cascade [14]. Upon leptin engagement, receptor-associated JAKs become activated and phosphorylate each other as well as the intracellular tail of their receptors, thereby creating docking sites for STATs directly which leads to their DNA binding and target gene activation [19,20]. Overall, leptin binding to OB-R activates JAK/STAT, a protein tyrosine kinase, and mitogen-activated protein kinase (MAPK) signaling pathways [21]. JAK-mediated phosphorylation activates STATs, which in turn directly bind DNA and regulate gene expression [22].

More specifically, upon leptin binding, OB-R homodimerizes and signals via phosphorylation of JAK2 and STAT3 in both benign and malignant mammary cell lines, which activates the MAPK extracellular signal-activated kinase 1/2 (ERK1/2) [7]. However, the OB-R/leptin homodimer model has been questioned; therefore, a higher order clustering OB-R model has been proposed [20,23]. Leptin-regulated signaling comprises both canonical and non-canonical pathways commonly triggered by multiple cytokines. The canonical signaling pathway involves activation of JAK2/STAT, MAPK/ERK 1/2 and PI-3K/AKT1, while the non-canonical pathway activates PKC, JNK, and p38 MAP kinase [24,25]. Effective communication between cells is central to development of tissue and organism homeostasis as each of these leptin-regulated signals is vital due to their biological effects on food intake, adiposity, energy equilibrium, endocrine and immune systems, and oncogenesis [24,25].

Although several leptin receptor isoforms exist, OB-Rb (L) is the only one containing an intact intracellular domain capable of inducing the intracellular JAK/STAT pathway upon ligand binding [1]. OB-R extracellular domain (816 amino acid residues) is found in all isoforms. However, variable lengths of the intracytoplasmatic domain (300 amino acid residues) differentiate OB-R isoforms [26]. The long isoform of OB-R (OB-RL or OB-Rb) shows the JAK-STAT (Box 2) and PI-3K and MAPK (Box 1) docking sites and, thus has full signaling capabilities. Compared to similar observations in other species, leptin stimulation of murine OB-RL involves phosphorylation of three specific tyrosine residues (Tyr985, Tyr1138, and Y1077) in the C-terminal domain [6]. Phosphorylated Tyr1138 of OB-R serves as a binding site for STAT proteins. Binding of SH2 domain-containing protein (i.e., STAT3) to Tyr1138 stimulates the main leptin-signaling pathway in the hypothalamus [6,27]. In addition to Tyr1138, Tyr985 in the OB-R intracellular domain was suggested to be involved in JAK-STAT3 activation [28,29,30]. Phosphorylated Tyr residues on JAK2 interact with SH2 domains of SOCS to regulate OB-Rb signaling [1]. Binding to the Y1077 site occurs at a significantly lower efficiency, possibly suggesting an accessory role for this site [6,25]. SOCS-3 binds Tyr 985 of OB-R and inhibits its signaling [28,30]. OB-R isoforms with shorter intracytoplasmatic domains have only Box 1, thus lacking JAK/STAT signal activation motif. It is believed that OB-R isoforms are derived from mRNA alternative splicing [28,30]. Leptin signaling also results in STAT3 binding, although STAT1, STAT5 and STAT6 may be activated by leptin as well [1,31,32].

The STAT3 pathway is essential for mediating leptin actions on body weight, appetite and glucose metabolism [29]. Various studies have demonstrated that the STAT3 pathway is strongly influenced by leptin action in proliferation [33,34], migration [35], and anti-apoptosis [36] of malignant cells. Leptin-induced activation of STAT3 regulates several genes involved in cancer, which include cyclin D1 [6,33,34], cyclooxygenase (COX)-2 [37], VEGF [6,7], human telomerase reverse transcriptase (hTERT) [15], Survivin [38] and leptin [39]. In addition, leptin activation of STAT3 upregulates IL-1 [40] and Notch [25] in breast cancer and markers of cancer stem cells in pancreatic and breast cancer [8].

Notch, IL-1 and leptin are known pro-angiogenic inducing factors in breast cancer [25]. Notch signaling and crosstalk with multiple signaling pathways have an essential role in breast cancer cell proliferation, migration, invasion, metastasis and even angiogenesis [25,41]. Leptin up-regulates Notch1-4/JAG1/Dll-4, while Notch targets genes, Hey2 and Survivin, together with IL-1 and VEGF/VEGFR-2 in breast cancer cells. RNA knockdown and pharmacological inhibition of leptin signaling significantly abrogated activity of reporter gene-luciferase CSL (RBP-Jk) promoter, indicating that it was linked to leptin-activated JAK2/STAT3, in addition to other canonical leptin signaling pathways [24]. These data strongly suggest that leptin induces Notch in breast cancer through JAK2/STAT3. Leptin significantly influenced proliferation/migration along with VEGF/VEGFR-2 that was highly dependent on a novel unveiled crosstalk between Notch, IL-1 and leptin (NILCO) in breast cancer cells [6,24,25].

Synergy of leptin/STAT3 with HER2 receptor induces tamoxifen resistance in breast cancer cells through regulation of apoptosis-related genes [42]. STAT3 phosphorylation was increased by leptin and tamoxifen in MCF-7 compared with TAM alone [42]. Additionally, epithelial-mesenchymal-transformation (EMT) and migration of endothelial cells were dependent of leptin activation of STAT3 via Snail/vascular endothelial cadherin-independent mechanism [43]. Moreover, leptin-induced STAT3 phosphorylation increased stemness in embryonic and tumor tissues, which was regulated by the feedback actions of pluripotency-associated transcription factors (i.e., NANOG, OCT4, SOX2) [44].

**Figure 1:**
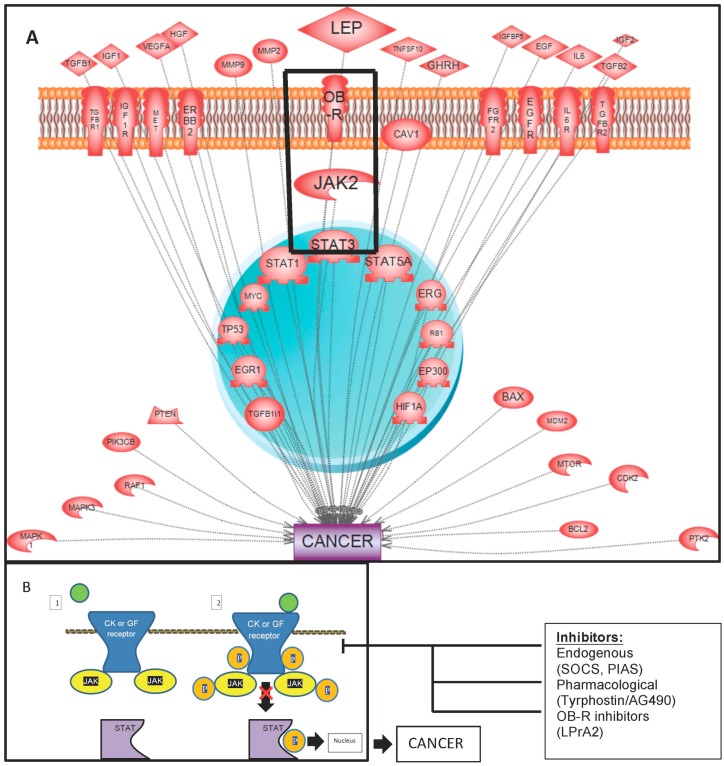
Leptin and jak-stat in cancer. (**A**) JAK2/STAT3 signaling and crosstalk in cancer. Key regulators of the JAK/STAT pathway are shown via activation or inhibition as identified by the Pathway Studio program (Ariadine Genomics, Rockville, MD, USA), which shows extensive crosstalk between molecules. Crosstalk and convergence of different signaling pathways are necessary for STAT activation of DNA-binding or transcriptional activity. The JAK/STAT pathway can regulate a plethora of processes including apoptosis, cell differentiation, and angiogenesis, all of which are associated with cancer growth and metastasis. The leptin-induced JAK2/STAT pathway plays an important role in obesity-related cancers. A detailed description of relationships detected is included in the supplementary material; (**B**) The JAK-STAT signaling pathway. Upon binding ligand to CK or GF receptor, the receptor-associated JAK becomes activated and mediates JAK phosphorylation while also phosphorylating the intracellular tail of the receptor. This leads to the recruitment of specific STATs, which are then also activated through phosphorylation. Activated STATs are released from the receptor, dimerize, translocate to the nucleus, and bind to the DNA-promoter regions of target genes.

Leptin activation of JAK/STAT and other canonic pathways induce several processes involved in cancer. However, a main feature of leptin signaling pathway is its crosstalk with several oncogenic signaling pathways that confer survival advantage to rapidly proliferating cancer cells (see Figure 1; Supplementary Table S1).

## 4. Leptin and Insulin

In light of considerable evidence that key metabolic hormones physiologically regulate energy balance and glucose homeostasis, it is suggested that the ultimate actions of leptin and insulin signaling networks are interconnected in multiple tissues [45]. Leptin/OB-R-induced JAK2 activation triggers tyrosine phosphorylation of STAT3, STAT5b, (insulin receptor substrate -1 and -2 (IRS-1 and IRS-2) [46]. However, insulin was more effective than leptin in stimulating IRS phosphorylation. This indicates that there is a positive crosstalk between insulin and leptin signaling pathways at the level of JAK2 and STAT5b in rat liver [46].

Simultaneous treatment with both hormones yielded no change in maximal phosphorylation of STAT3, interferon regulatory sequence (IRS)-1, IRS-2 and Akt, but led to a marked increase in tyrosine phosphorylation of JAK2 and STAT5b when compared with isolated administration of insulin or leptin [46].

## 5. IGF-1 and Leptin

Insulin-like growth factor-1 (IGF-1) is a critical growth factor for various types of cells [36]. Challenge of target cells with IGF-1 results in a pleiotropic response that can include cellular proliferation or differentiation, stimulation of amino acid uptake, glycogen metabolism, and induction of mRNA and protein synthesis [47]. In addition to the canonical IGF-I signaling pathways through extracellular-regulated kinase (ERK) and phosphatidylinositol-3 kinase (PI3K)-Akt, IGF-I also signals through the JAK/STAT pathway as activation of this pathway may lead to induction of suppressor of cytokine signaling (SOCS) molecules [48]. SOCS are important negative regulators of the JAK/STAT pathway and serve a pivotal role in preventing the growth and proliferation in cancer cells. IGF-I stimulates the activation of STAT-1 and STAT-3 in several cell types [36,49,50,51]. It was reported that a novel bidirectional crosstalk between IGF-I and leptin signaling occurs in breast cancer cells [34]. Both ligands, IGF-I and leptin were found to be able to induce phosphorylation of IGF-1 and OB-Rb, respectively, which induced synergistic activation of downstream effectors, Akt and ERK 1/2. These leptin effects were found in parallel with the increased phosphorylation IRS-1 and IRS-2 [34].

## 6. Leptin, C-Src, Grb2 and EGF

Human cellular-Src (c-Src) is one of the key-signaling node for STAT3 and STAT5 activation in normal as well as malignant epithelial cells, and recent studies have shown that activation of this non-receptor tyrosine kinase has been detected in breast cancer cell lines and primary breast tumor specimens [12,15]. When activated by leptin or estrogen in breast cancer cells, STAT5 requires c-Src and epidermal growth factor (EGF). However if the EGF receptor (EGFR)/c-Src pathway is hyperactivated, the influence of this oncogene could enhance tumor cell proliferation in addition to potentially promoting chemo-resistance [25]. Leptin and IGF-I synergistically transactivate EGFR [52]. Furthermore, it was shown that leptin induces the phosphorylation of HER2 partially abrogated by inhibition of JAK via the pharmacological inhibitor AG490 [52].

The JAK/STAT pathway utilizes a group of essential elements in addition to cytokine receptor polypeptides to induce activation [16]. This family of SH2 domains contains STATs and, along with two flanking SH3 domains, makes up the adaptor protein, Grb2 [53]. The canonical model of Grb2 function relies on the widely-confirmed observation that Grb2 is constitutively associated with Son of sevenless (SOS) and upon growth factor activation and tyrosyl phosphorylation, Grb2 brings Sos1 into close proximity of membrane-bound Ras, thereby activating Ras and the downstream MAPK cascade [54]. Grb2 signaling is critical for cell cycle progression and actin-based motility, and, consequently, more complex processes, such as epithelial morphogenesis, angiogenesis and vasculogenesis [54]. It was earlier reported that leptin induces phosphorylation of SH2-containing protein SHC in human embryonic cells (HEK 293) that, in turn, is associated with the adaptor protein, Grb2. Phosphorylated SHC-Grb2 could be linked to Ras activation and may be a critical step in proliferation and/or differentiation of cells [55]. Leptin-induced JAK2-Gbr2 activation was linked to neuroprotective effects. Moreover, knockdown of the GRB2 prevented leptin-induced pERK1/2 activation and neuroprotection. Leptin/pERK1/2 induces CREB phosphorylation and nuclear localization, which is a survival factor for dopaminergic neurons [56]. Moreover, in breast cancer, leptin induces Src/Gbr2/Gab2/STAT3 activation and Rac-1 crosstalk to facilitate VEGF/VEGFR2 activation [25].

## 7. Leptin, HGF and c-Met

Hepatocyte growth factor (HGF) and its receptor, C-Met (an oncogene), are both critical mediators of breast cancer progression and are highly expressed in these tissues [57]. Induction of epithelial tubules by growth factor HGF depends on the STAT pathway [58]. Grb2 is activated during HGF/C-MET signaling, leading to the activation of downstream MAPK pathway involved in the cellular proliferation and differentiation, and can also be involved in cellular invasion and motility through activation of downstream focal adhesion kinase (FAK) pathway [57]. There are contradictory reports concerning the effects of leptin on mammary epithelial cells. It was reported that leptin induces morphogenesis and proliferation of mammary cells. However, it was also shown that leptin affected HGF-induced mammary morphogenesis via unknown mechanisms in mammary epithelial cells from bovine origin [59]. Baseline leptin levels/BMI ratios were positively correlated with baseline levels of HGF and other angiogenic factors in patients with CAD (leptin/BMI ratio vs. HGF: *r* = 1.07, *p* < 0.01 [36].

## 8. Leptin, IFN-γ and IFN-α

Interferon (IFN)-receptor interaction at the cell surface leads to the activation of kinases of the JAK family that then phosphorylate STATs, which translocate to the nucleus where they bind to specific sequences (DNA response elements) and direct transcription [60]. IFN receptor consists of two subunits, IFNAR1 binds Tyr2 and IFNAR2 binds JAK1 [41]. Activation of JAK-1 and -2 by IFN results in the coordinated phosphorylation and activation of STAT signaling proteins, specifically STAT1, and to a lesser extent, STAT3 [61,62]. JAK1 and TYK2 are activated by receptor engagement to phosphorylate the intracellular domains of the IFNα/β receptors, which provide recruitment sites for the latent STAT1 or STAT2 SH2 domains [63]. At these sites, phosphorylation of STAT1 and STAT2 primarily drives dimerization and formation of the interferon stimulated gene 3 (ISGF3) complexes [63]. Interferon-alpha (IFN-α) significantly suppresses leptin secretion in adipose tissues [64].The element responsible for the IFN-α response is a highly conserved region of 12–15 base pairs, the interferon stimulated response element (ISRE), while IFN-γ causes immediate transcriptional activation of a consensus immediate response element, the IFN-γ activation site (GAS) [60]. Interestingly, leptin, an inflammatory cytokine, induces the expression of IFN-γ-inducible nitric oxide synthase (iNOS) and cyclo-oxygenase-2 (COX-2), both prominent markers of macrophage activation [65]. Additionally, when leptin is released following an inflammatory state, it can induce co-operation with IFN-γ, nitric oxide (NO), and prostaglandin E2 (PGE2) release, contributing to sustaining the ongoing inflammatory response [65]. Leptin can induce Th1 phenotype in mice via increased synthesis of IFN in stimulated T lymphocytes. Moreover, leptin can affect anti-CD3 stimulation of T cells and secretion of IFN [66].

## 9. Leptin and IL-1

Leptin regulates inflammatory cytokines, including interleukin-1 (IL-1), in diverse tissues and pathological conditions [33,67] in which both cytokines signal through JAK/STAT3. Because both leptin and IL-1 are inflammatory and proangiogeneic factors that upregulate VEGF, the association between IL-1 and leptin could be a critical event for tumour angiogenesis [6,51]. Furthermore, studies have shown the blockade of IL-1 receptor partially abrogated leptin-mediated increase of both VEGF and VEGFR2 protein and mRNA, strongly suggest that leptin pro-angiogenic signature in breast cancer could partially be mediated by IL-1 signaling [6,40]. IL-1 upregulation involves leptin activation of JAK2/STAT, PKC, p38, MAPK/ERK1/2, PI-3K/AKT1 and JNK suggesting that multiple leptin-induced signaling pathways can affect leptin-IL-1 crosstalk in breast cancer [40]. These cytokines could actively crosstalk in breast cancer eliciting pro-inflammatory and proangiogenic effects that contribute to cancer growth. Leptin increases protein and mRNA levels of all components of the IL-1 system implying that leptin proangiogenic activity involves JAK2/STAT3 activation [6].

## 10. Leptin, ATM and IL-6

Adipose tissue macrophages (ATMs) are associated with insulin resistance in a manner that is dependent upon their activation status. However, more recent studies imply that ATMs may have housekeeping functions in adipose tissue and could provide physiological roles in modulating lipid flux in adipocytes [68]. Interleukin 6 (IL-6) is a cytokine secreted from ATMs that signals via the activation of JAKs and transcription factors of the STAT family. All IL-6-type cytokines recruit membrane glycoprotein 130 (gp130) to their receptor complexes, which signal to activate JAKs and to recruit STAT proteins due to IL-6 inducing gp130-homodimerization [24,69]. Upon stimulation, gp130-associated kinases Jak1, Jak2, and Tyk2 become activated, phosphorylating the cytoplasmic tail of gp130, which is important because several phosphotyrosine residues of gp130 are docking sites for STAT factors with matching SH2 domains, mainly STAT3 and STAT1 [70,71]. In microglia, leptin increased IL-6 production via the activation of IRS-1/PI3K/Akt/NF-kB and p300 signaling pathways [72]. Similarly, in human synovial fibroblasts, leptin was identified as an inducer of IL-6 [73]. These relationships do not seem to be univocal, as it was reported that IL-6 regulates the expression of leptin production by adipocytes [74]. The result of IL-6-type cytokine signaling is due to the regulation of a variety of intricate cellular processes, such as proliferation, differentiation and gene activation, which when deregulated is associated with angiogenesis and metastasis through fueling STAT3, MAPK, and Akt signaling [25,71].

## 11. Inhibition of the JAK-STAT Pathway and Cancer

In addition to adipose tissue, leptin is expressed and secreted by some cancer cell types. OB-R is mainly expressed in the hypothalamus and adipose tissue, but weakly expressed in peripheral cells. In contrast, OB-R is overexpressed in cancer cells [41]. Current data suggest that leptin shows strict specificity for binding to OB-R and vice versa. It is a fact that leptin can induce cancer progression and tumor angiogenesis. Leptin could play an important role in the rise of obesity-related cancer incidence [8]. Therefore, leptin/OB-R signaling could be an attractive target for cancer prevention and therapy. Several antagonists of leptin signaling have been reported for cancer treatment [10,20,22,32,61]. We have designed and tested several inhibitors of leptin/OB-R signaling, named leptin peptide receptor antagonists (LPrA) [6]. Accumulated evidence suggests that LPrA are effective inhibitors of leptin signaling and potential novel adjuvants for cancer treatment [7]. An additional way to abrogate leptin signaling in cancer is by targeting JAK/STAT pathway.

It is imperative to identify factors that can inhibit the JAK/STAT pathway because hyperactivation can promote tumor growth and induce inflammation as well as regulate other genes that control cell proliferation, differentiation, tumor development, and cell survival [75]. While STAT3 signaling in transformed epithelial cells promotes tumor growth by increasing response stimuli in pro-survival and cell cycle genes, STAT1 is known to suppress tumorigenesis through its function in regulating IFNγ [65]. STAT1 and STAT2 are key mediators of type I and type III IFN signaling, which associates with IFN regulatory factor 9 (IRF9) to form a heterotrimeric transcription factor complex responsible for activating STAT transcriptional regulation [63]. STAT-induced effects on targeted genes could have therapeutic implications. Modulation of STAT signaling could potentially serve as adjuvant target to chemotherapy, mainly because STAT-activated signaling pathways can control and alter the growth of cancer cells [76]. This would create a manageable regimen that is less toxic to healthy cells [76].

## 12. SOCS

The eight member SOCS family (SOCS1-7 and CIS) are important regulators of cytokine-mediated signaling and are characterized by an amino terminal region of limited homology, a central SH2 domain and conserved SOCS box at the C-terminus [11,12,45]. SOCS1 is a critical tumor suppressor in specific cancers [11,12,45]. Recent studies have shown SOCS1, SOCS2 and SOCS3 genes to be abundantly expressed in the ductal and alveolar epithelia of the developing mouse mammary gland [45]. SOCS3 expression is activated by leptin and serves as a negative regulator of leptin signaling as it binds directly to the JAKs. By binding to phosphorylated Tyr 985 of OB-Rb, overexpressed SOCS suppresses leptin-induced tyrosine phosphorylation of JAK2 and ERK activation. With SOCS3 role as a prominent negative regulator of both leptin and insulin signaling, it has been implicated in the pathogenesis of obesity as well as associated metabolic abnormalities [45]. The SOCS proteins are rapidly induced by cytokines, and act as negative feedback regulators of cytokine signaling through several mechanisms, including direct binding to tyrosine-phosphorylated JAK or cytokine receptors and proteosomal degradation of signaling proteins via SOCS box-mediated ubiquitination complex [45].

## 13. TGF-Beta

TGF-beta signaling carries out a dual role during the progression of cancer. One of its key functions is to maintain homeostasis of several cell types like epithelial, endothelial, and hematopoietic cells, therefore acting as a tumor suppressor in the early stages of cancer through the induction of cell cycle arrest and apoptosis [77]. Later in cancer development, due to oncogenic mutations in this pathway, TFG-beta becomes capable of promoting tumor growth and metastatic functions via epithelial to mesenchymal transition induction [77]. Interestingly, studies have shown that TGF-β downregulates IL-2 and IFN-γ in CD4+ cells, which is significant to the JAK/STAT pathway because of the role of this interferon in transcription activation [78,79]. TGF-β is one of several inflammatory cytokines that can modulate the synthesis and secretion of leptin from adipose and tumor cells, thus linking leptin with the inflammatory response [6,68].

## 14. PTPN9

Protein tyrosine phosphatase, non-receptor type 9 (PTPN9) is a soluble tyrosine phosphatase that attenuates prolactin- and EGF-mediated STAT5 activation, which regulates expression of genes that promote cell survival and proliferation in breast cancer cells [12]. The levels of phosphorylated EGF also are enhanced upon inhibition of PTPN9-mediated by MicroRNA miR-24 [80]. Studies have shown that PTPN9, in addition to other prototypical tyrosine phosphatases, can down regulate insulin and leptin signal transduction [80].

## 15. Caveolin-1

Adipose tissue has an extremely high abundance of caveolae with adipocytes having a higher concentration of this protein than any other cell type [77]. The caveolin-1 is a member of a family of 21–24 kDa integral membrane proteins that, in addition to binding cholesterol and fatty acids, are responsible for maintaining the structure of caveolae [77]. Caveolin-1 clearly plays critical roles in cholesterol transport, endocytosis, and signal transduction [81]. It is thought to function as a scaffolding protein that organizes and concentrates cholesterol, glycosphingolipids, and caveolae associated signaling molecules, such as endothelial nitric oxide synthase [24]. In mammalian cells, caveolin-1 protein has been reported to inhibit the JAK/STAT signaling pathway through direct interaction with STAT3 protein as reviewed by Guo et al, 2011 [26]. Leptin mediated proatherogenic mechanism that was linked to a novel caveolin-1 dependent feedback, which may be related to the development of peripheral leptin resistance in the endothelium [82]. Interestingly, caveolin-1-deficient mice are resistant to diet-induced obesity, because of reduced/atrophic fat deposits [83].

## 16. PIAS

Protein inhibitors of activated stats (PIAS) represent a class of negative regulators of the JAK/STAT pathway. The PIAS proteins bind to activated STAT dimers, consequently preventing them from binding DNA [84]. These proteins have centrally located Zn-binding RING-finger domain, a well-conserved SAF-A/Acinus/PIAS (SAP) domain at the N-terminus, and a less conserved carboxyl domain [10]. Recent studies indicate that PIAS proteins are associated with the E2 conjugase Ubc9. Moreover, PIAS proteins have E3 conjugase activity, which is involved in sumoylation mediated by the RING finger domain [85]. Leptin signaling in specific cells in the hypothalamus actually induce the expression of gene encoding PIAS [10,84,85].

## 17. Highlights

Obesity is strongly linked to the development of several cancer types. Current data suggest that leptin signaling could be an essential link between obesity and cancer incidence and development [6]. Leptin-induced effects in cancer are linked to the activation of JAK/STAT signaling pathway, which is involved in the upregulation of Cyclin D1 and proliferation of cancer cells [8,41]. High levels of pro-angiogenic factors, leptin, IL-1, Notch and VEGF (ligands and receptors), are found in breast cancer and commonly correlated with metastasis [41]. Leptin upregulation of VEGF/VEGFR-2 is mediated by leptin-induced JAK/STAT3-Notch expression while also showing that leptin regulation of VEGF/VEGFR2 in breast cancer involves the activation of Src and Gbr2/Gab2/STAT3, suggesting crosstalk with Rho-GTPases. Recent studies have shown that leptin-induced JAK/STAT3 activation and Notch collaborate to promote cell growth and migration and that a complex signaling network between Notch, IL-1 and leptin (NILCO) is essential for upregulation of proangiogenic factors (e.g., VEGF/VEGFR-2) in breast, endometrial and pancreatic cancer. Furthermore, NILCO could be actively involved in leptin-induced cancer stem cell maintenance and drug resistance to chemotherapeutics [8]. Overall, leptin regulation of JAK/STAT signaling has biological consequences during pathway hyperactivation in cancer and becomes further complicated by leptin-signaling interactions with other signaling pathways [13,23,36,66,86].

Currently, several inhibitors of JAK/STAT signaling pathway are being tested in clinical trials (see Table 1). JAK inhibitors showed different degrees of efficacy in clinical settings, but drug resistance developed to specific treatments is still a concern [87]. It is important to determine which of the most promising regulators is most suitable for systemic delivery in a clinical setting. Furthermore, due to advances in genetic mapping, a unique combination of inhibitory compounds that target the JAK/STAT pathway at different points should be developed by a team of researchers/clinicians with the idea of creating a therapeutic regimen that is personalized to each individual patient’s genome.

The use of quantitative gene expression assays, such as ChIP-seq, along with nucleosome profiling and proteomic approaches, will provide greater insight into the current understanding of differential regulation of JAK/STAT target genes [63]. Additionally, as technology continues to evolve and methods based on chromatin conformation capture become more utilized, researcher may be able to examine beyond the local DNA-protein and protein-protein interactions and discover STAT interactions at distal regions [63]. STAT3 is part of a pathway that is critical for facilitating leptin actions on dietary intake, weight gain, and glucose metabolism; however, elevated levels have been correlated with poor prognosis among cancer patients [19,92]. In view of the prevalence of JAK/STAT3 hyperactivation in human cancers, and the potential role of leptin in these events, selective targeting of these proteins in cancer and cancer stem cells holds promise for significant advancement in the treatment of cancer [93].

## 18. Future Directions

JAKs phosphorylate STATs resulting in STAT-dissociation from the receptor chain, formation of homo- and heterodimers, and translocation into the nucleus where they serve as transcription factors. Various inhibitors of JAK family members have been developed and are currently used therapeutically [11,17,87]. Additional studies in both cultured adipocytes and in adipose tissue will be needed to reveal comprehensive roles of the JAK/STAT family members in adipocytes, obesity, and insulin resistance [13]. Although tyrosine phosphorylation is essential for canonical STAT activation, other covalent modifications, such as serine phosphorylation, acetylation, methylation and sumoylation, can also occur, and studies of these STAT modifications have the potential to provide useful insight regarding the regulation of this complex mechanism [94]. Current data suggest that inhibitors of leptin/JAK/STAT signaling could provide novel therapeutic strategies for cancer. It is envisaged that intense investigations in adipocyte biology, tumor microenviroment and obesity-related cancer will lead to the identification of new therapeutic targets for cancer and metabolic diseases.

## Figures and Tables

**Table 1:** Pharmacological inhibitors of the leptin-induced JAK/STAT pathway.

Pharmacologic Inhibitor	Targets	Diseases	Reference
Tofacitinib (Xeljanz)	Inhibition of JAK1 and JAK3 signaling	Ulcerative colitis, Crohn’s disease Ulcerative colitis	[87]
ZM39923 (ZM)	ZM has been described as the most specific JAK3 inhibitor	Rheumatoid arthritis	[88]
Tyrphostin/AG490 (AG)	JAK2 and JAK3 signaling	Rheumatoid arthritis	[88]
Ruxolitinib (JAKafi)	Potent inhibitor of JAK1 and JAK2 signaling	Solid tumor, metastatic pancreatic cancer Lung adenocarcinoma, metastatic breast cancer metastatic prostate cancer, NSCLC, breast cancer	[11,87]
Pyridone 6	Binds to the ATP pocket of the active conformation of JAK2	Myelofibrosis	[89]
CYT387	Inhibition of JAK2 signaling	Myelofibrosis	[90]
AZD1480	Inhibition of JAK2 and JAK1 signaling	Gastric cancer, hepatocellular carcinoma, metastatic lung cancer, NSCLC, solid tumor	[11,91]
Momelotinib	Inhibition of JAK1 and JAK2 signaling	Lung cancer, colon cancer, pancreatic cancer Metastatic pancreatic cancer, pancreatic ductal adenocarcinoma	[11,87]
INCB-39110	Inhibition of JAK1 signaling	Adenocarcinoma, solid tumor, metastatic pancreatic cancer	[11,87]
Peficitinib	Inhibition of JAK1 and JAK3 signaling	Ulcerative colitis	[87]

Over the past few years, several different pharmacologic inhibitors have been identified, providing processes by which cytokine signaling can be attenuated through the JAK/STAT signaling pathway. Negative regulation of signal transduction is essential for an appropriate cellular and physiological response to cytokine stimulation.

## Supplementary Materials

The following are available online at http://www.mdpi.com/2076-393X/4/3/26/s1, Table S1: Detailed description of relationships detected between leptin and the JAK/STAT pathway in cancer using Pathway Studio 9 (Ariadine Genomics, MD).

## Abbreviations

The following abbreviations are used in this manuscript:

AKT	serine threonine protein kinase 1
BCL2	B-cell lymphoma 2
CDK	cyclin dependent kinase
CK	cytokine
EGF	epidermal growth factor
EGFR	epidermal growth factor receptor
EP300	E1A binding protein P300
EPAS	endothelial PAS domain protein 1
ERBB2	erb-b2 receptor tyrosine kinase 2
ERG	v-ets avian erythroblastosis virus E-26 oncogene homolog
ETS1	v-ets avian erythroblastosis virus E26 homolog 1
FGF	fibroblast growth factor
FGFR	fibroblast growth factor receptor
GF	growth factor
GHRH	growth hormone releasing hormone
GRB2	growth factor receptor-bound protein 2
HGF	hepatocyte growth factor
HIF1A	hypoxia inducing factor 1A
IGF	insulin growth factor
IGFR	insulin growth factor receptor
IL-8	interleukin 8
LEP	Leptin
LPrA2	leptin peptide receptor antagonist
MAPK	mitogen activated protein kinase
MET	proto-oncogene, receptor tyrosine kinase
MMP	matrix metalloproteinase
MTC	v-myc avian myelocytomatosis viral oncogene homolog
MTOR	mammalian target of rampamycin
NOS	nitric oxide synthase
OB-R	leptin receptor
PIAS	protein inhibitor of activated stats
PIK3CB	phosphatidylinositol-4,5-bisphosphate-3 kinase, catalytic subunit beta
PGF	placental growth factor
PTEN	phosphate and tensin homolog
RAS	raf1, proto-oncogene, serine/threonine kinase, ras viral oncogene homolog
RB	retinoblastoma 1
RHOA	ras homolog family member A
SHC1	(src homology 2 domain containing) transforming protein 1
SOS1	son of sevenless 1
SRC	proto-oncogene, non-receptor tyrosine kinase
TGFA	transforming growth factor A
TP53	tumor protein 53
VEGF	vascular endothelial growth factor
